# American Antidote against Snake-Poison

**Published:** 1851-02

**Authors:** 


### MATERIA MEDICA AND THERAPEUTICS.

*American Antidote against Snake-Poison.*—Dr. Pereira submitted to the meeting a specimen of cedron seeds (seeds of *simaruba cedron*) from Panama, in South America. These seeds have long been used, and with great confidence, by the native doctors of Panama, as an antidote against snake-poison. The reputation they have thus acquired has recently attracted attention in France, where it is said a great congress of medical men is appointed to be held next month, for the purpose of testing, by experiment, the virtue of this celebrated remedy, and two gentlemen, M. Auguste Guillemin and M. Hippolyte Fournier, Professor of Mathematics of the department of Arvegron, have offered themselves to be operated upon. It is announced that all the different States of Europe will be represented at the congress. The remedy is proposed to be applied to the cure of mental disease and epilepsy. Notwithstanding the interest which has thus been excited on the Continent, by the description given of the wonderful effects produced by the *cedron*, Dr. Pereira appears to have very little faith in its efficacy, at least in such cases as those referred to.—*London Medical Times, Dec.* 14, 1850.

[In addition to the statement given above, we have received a letter addressed to Dr. Peter Smith of the City Hospital at San Francisco, California, by Don José de Obaldia, Governor of Panama, dated Nov. 22d, 1849, in which he confirms the experience of the native physicians of Panama. He says that the tree which produces the fruit, and is called the "cedron," is described in the scientific work of Dr. Cespedes, one of their own naturalists, and that the fruit is given internally in five grain doses, as an antidote to the bite of the rattlesnake, viper, and scorpion, and to the sting of wasps, &c.

For internal use, the scrapings of the fruit are administered in a wine-glassful of spirits or warm water, at the same time that the wound is washed in an infusion and covered with the scrapings, secured by a bandage. The dose is to be repeated every two hours till relieved. To quench the attendant thirst, a weak infusion of the cedron is exhibited.

Several cases are related in which the cedron proved beneficial, where other means had failed to restore those bitten by venomous snakes, and what is worthy of notice, the recovery was much more rapid than in the case of those less violently affected and who were restored by other means.

The cedron has also been used successfully in cases of retained placenta (Pares,) both as an internal remedy and an external application to the genitals.

The tree grows abundantly in humid places, and as the wood is inapplicable to the uses of life, and the fruit rejected by animals on account of its bitterness, an abundant supply is predicted. We are also informed that the scrapings have been very sucessfully employed by Dr. P, Smith as a substitute for Quinine in the treatment of malarious fevers. A drawing of the fruit will be found accompanying Dr. Horner's article on California in this number.—Eds. Examiner.]

